# Ovariectomy in mouflons under field conditions: a comparative assessment of midventral and flank laparotomy approaches

**DOI:** 10.3389/fvets.2024.1405847

**Published:** 2024-06-19

**Authors:** Lorenza Frattina, Alice Carbonari, Matteo Burgio, Francesca Giannini, Francesco Locantore, Annalisa Rizzo, Vincenzo Cicirelli

**Affiliations:** ^1^Department of Veterinary Medicine, University of Bari Aldo Moro, Valenzano, Italy; ^2^Natural Park of the Tuscan Archipelago, Portoferraio, Italy

**Keywords:** mouflon, ovariectomy, laparotomy, midventral approach, flank approach

## Abstract

Ovariectomy is the best method to control the density of wild ungulate populations. The present study aimed to compare two surgical approaches of ovariectomy, via the flank and midventral, in mouflons under field conditions. A total of 20 female mouflons were enrolled in the study. The animals were divided randomly into two equal groups; group F animals were gonadectomized via the flank approach, while group L animals were sterilized via the linea alba access. The parameters evaluated were duration of surgery, duration of anesthesia, recovery time, intraoperative and postoperative complications, intraoperative nociception, and pain during the postoperative period. There were no intraoperative and postoperative complications. The evaluated parameters showed a very similar trend in both groups. Both techniques were found to be effective and safe in execution.

## 1 Introduction

The control of overabundant wildlife populations is of increasing concern to the public and wildlife managers due to the damage caused by wild animals on vegetation, ecosystems, and automobiles (1). Control measures for overabundant wild species has conventionally focused on lethal removal, a method rejected due to legal, safety, and ethical concerns (2). Alternative methods are studied, including translocation, predator reintroduction, birth control through contraception (3, 4), and surgical sterilization (5–7). The disadvantages of translocation include high costs, the exposure of animals to elevated stress during transport, increased risk of disease transmission, and difficulty in finding other release sites (8, 9). Predator reintroduction involves addressing security issues related to negative human–predator interactions (4, 10, 11). Birth control might reduce and maintain some animal populations at desired levels (12). Birth control can involve the use of drugs (temporary control) or surgical sterilization (permanent control) (13). It was reported that contraceptive drugs have limited success. Disadvantages are due to uncertainty in identifying treated individuals, the need for repeated treatment, high costs, and secondary consumption by non-target animals (e.g., scavengers), (14, 15). Surgical sterilization included tubal ligation or transection, ovariectomy, or ovariohysterectomy, which can be performed via laparotomy or laparoscopy. Tubal ligation or transection halts reproduction without altering normal hormonal function and then inducing a prolonged breeding season with prolonged mate-searching behavior (5, 12). The ovariectomy or ovariohysterectomy has been widely used in different wild species because these techniques are effective in reducing stress and costs associated with recapturing and administering the necessary doses of vaccines (1). The surgical technique can be performed using two approaches: flank and midventral, both of which are currently employed in the surgery of large animals (16). In ruminants, the flank approach is the most widely and frequently practiced, offering several advantages, such as the surgical site can be visualized and observed from a distance; it has a reduced potential risk for evisceration in case of wound dehiscence; and suturing the oblique muscles of the abdominal wall helps maintain its integrity (16). These advantages are important mainly for wild animals. The midventral approach is also employed in the surgery of large animals, but it is often performed in surgical facilities (7, 17). The existing literature does not show the differences between the two approaches; therefore, this study aimed to compare flank and midventral laparotomy approaches in the ovariectomy of mouflons under field conditions. To compare these approaches, we evaluated the time of surgeries, intraoperative or postoperative complications, intraoperative nociception, and postoperative pain. This study hypothesizes that there are differences in the tested parameters between the two approaches, aiming to determine the better surgical approach for ovariectomy performed in field conditions. Based on the literature, it is conceivable that the flank approach is better for these wild animals in terms of the duration of surgery and complications.

## 2 Materials and methods

### 2.1 Ethical consideration

The protocol for animal research was approved by the Ethics Committee for Animal Testing of the Department of Veterinary Medicine of the University of Bari “Aldo Moro,” Bari, Italy with the approval number 20/2023.

### 2.2 Animals

All mouflons were relocated from Giglio Island to the Marsiliana Nature Reserve for the project LIFE18NAT/IT/000828 LETSGO GIGLIO “Less alien species in the Tuscan Archipelago: new actions to protect Giglio island habitats”. All animals were transferred nearly 1 month before surgery. The mouflons were sexually mature (18–30 months) and in good health condition (body condition score of 3), without previous pathologies and were allocated to the very low anesthetic risk class (ASA 1). This study enrolled 20 female mouflons. The animals were randomly divided into two groups by drawing lots. In the first group (F), the animals were gonadectomized using the left flank as the surgical access, and the animals were gonadectomized using the linea alba access in the second group (L). For all animals, duration of surgery (from skin incision to the placement of the final suture), duration of anesthesia (from induction with propofol to the interruption of isoflurane administration), and recovery time (from the interruption of isoflurane administration to the recovery of quadrupedal station) were evaluated. To compare the two surgical techniques, intraoperative nociception (measured by the heart rate, respiratory rate, non-invasive blood pressure, and temperature), intraoperative and postoperative complications, and postoperative pain were evaluated.

### 2.3 Anesthetic protocol

Food was withheld from the animals for 24 h before gonadectomy. The day before the procedure, the mouflons were captured in a funnel-shaped enclosure. On the day of gonadectomy, the mouflons were captured from the enclosure by operators and contained for premedication (18). The premedication consisted of xylazine (Nerfasin^®^ 20 mg/mL, ATI, Ozzano dell'Emilia, Italy) at a dose of 0.1 mg/kg and a combination of tiletamine and zolazepam (Zoletil^®^ 50/50 mg/mL, Virbac, Milan, Italy) at a dose of 4 mg/kg. These drugs were mixed in the same syringe and injected into the brachiocephalicus muscle. To provide antibiotic coverage, 10.000 UI/kg benzylpenicillin and 12.5 mg/kg dihydrostreptomycin (Repen, Fatro S.p.A., Ozzano dell'Emilia BO, Italy; 200.000 UI + 250 mg/mL) were administered via an intramuscular (IM) injection 10 min after the premedication. A 20-G venous catheter (DeltaVen^®^, DeltaMed S.p.A., Viadana, Italy) was then inserted into the cephalic vein to initiate maintenance fluid therapy. The therapy included administering 3 mL/kg/h of Ringer's lactate, with possible adjustments during surgery based on the hemodynamic needs. Propofol (PropoVet Multidose^®^ 10 mg/mL, Zoetis Italia S.r.l., Rome, Italy) was administered intravenously at a dose of 2 mg/kg to facilitate orotracheal intubation. Anesthetic maintenance was performed using isoflurane (Isoflo^®^, Zoetis Italia S.r.l., Rome, Italy) in an open anesthesia system (MedVet S.r.l., Taranto, Italy) by the same operator. All patients were connected to a re-breathing respiratory circuit and allowed to breathe spontaneously. This protocol was followed according to the guidelines described by Caulkett and Haigh (19). Throughout the perioperative period, the animals' heart rate (HR), respiratory rate (RR), and non-invasive mean arterial blood pressure (MAP) were continuously monitored using multiparametric monitoring. If the parameters increased by more than 25% compared to the preincision values during the procedure in response to the surgical pain, a bolus of fentanyl would be administered intravenously at 2 μg/kg (Fentadon^®^, Dechra Veterinary Products S.r.l., Torino, Italy) as rescue analgesia. In addition, oxygen hemoglobin saturation (SpO_2_) and body temperature (T) were monitored using a pulse oximeter and a thermometer to ensure safe and controlled anesthesia.

### 2.4 Surgical procedure

**Figure 1:**
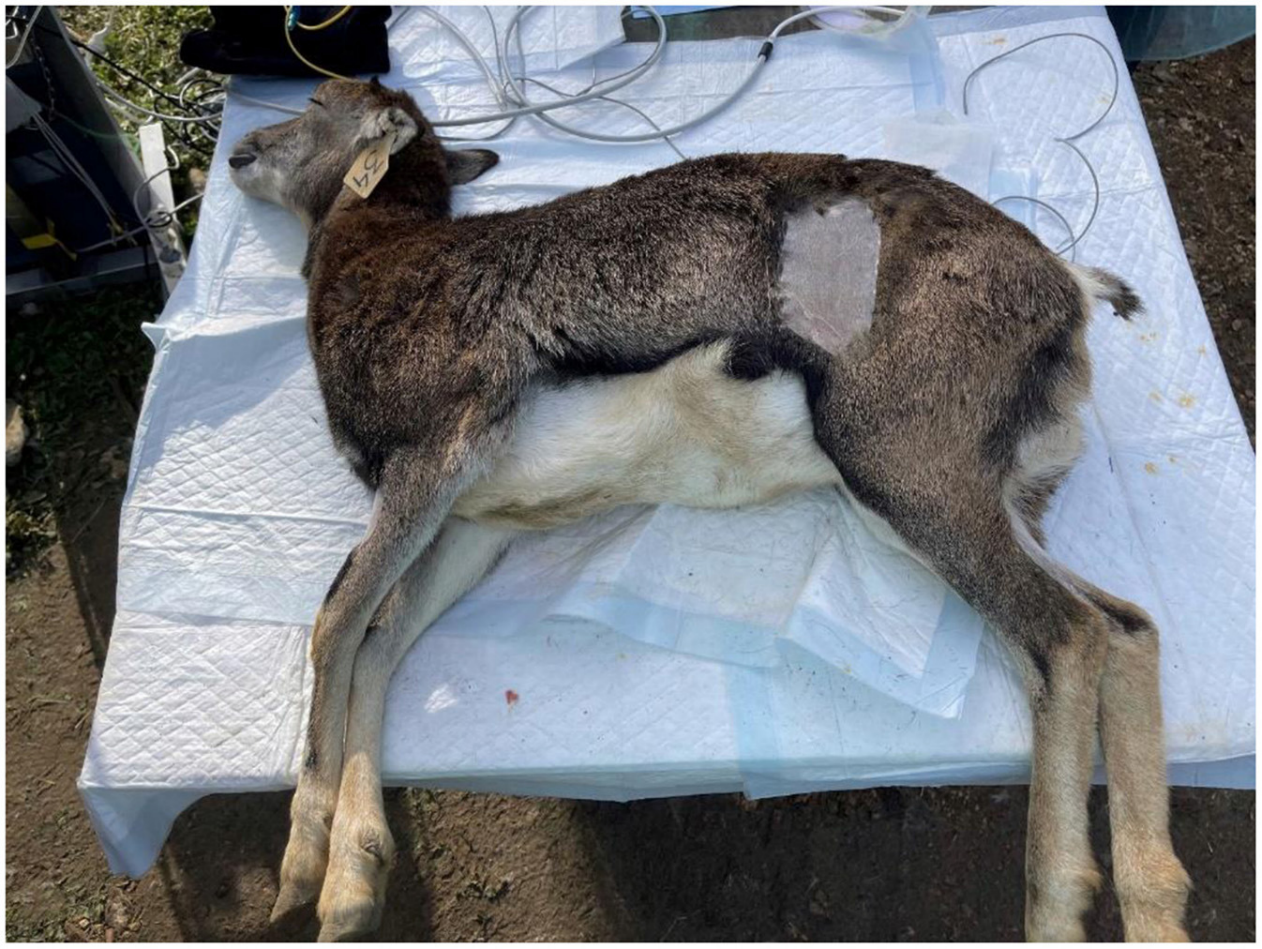
Surgical site to perform ovariectomy using the flank approach in a mouflon.

All surgical procedures were performed by the same surgeon staff. A total of 10 animals (group F) were gonadectomized via the left flank as the surgical access (Figure 1).

**Figure 2:**
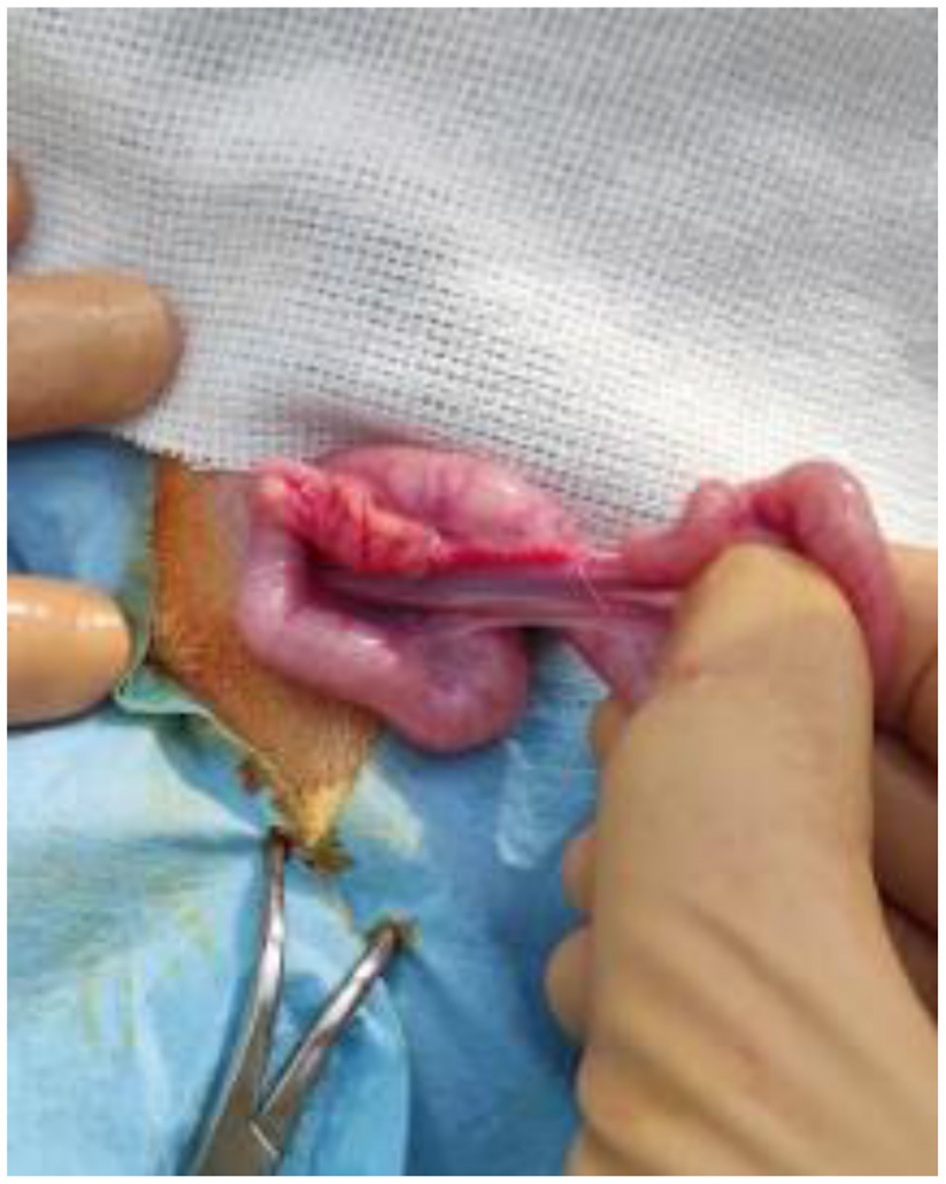
The uterus and the ovary of a mouflon exteriorized from the flank surgical site.

**Figure 3:**
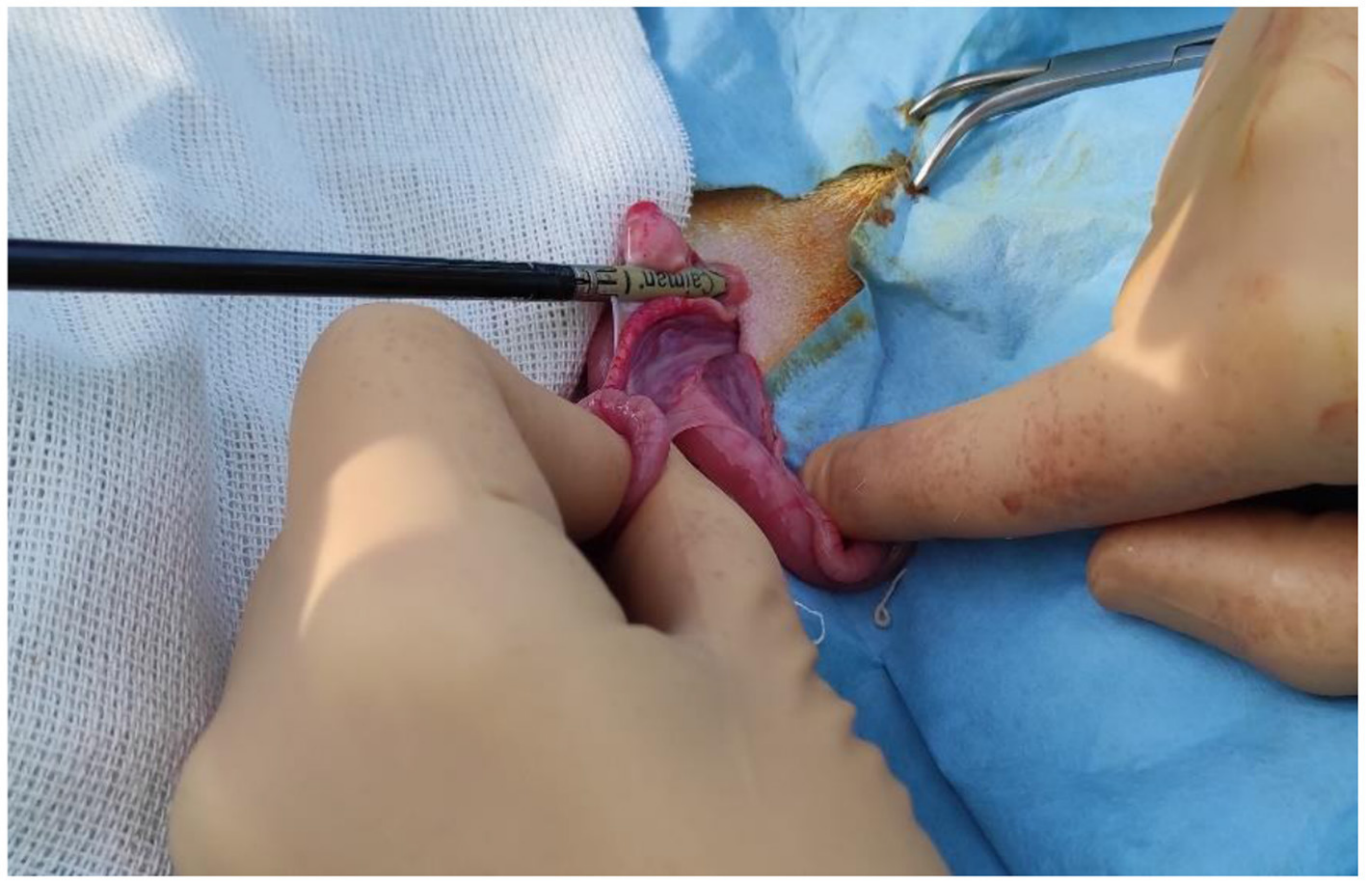
CAIMAN^®^ forceps placed at the base of the ovarian pedicle.

**Figure 4:**
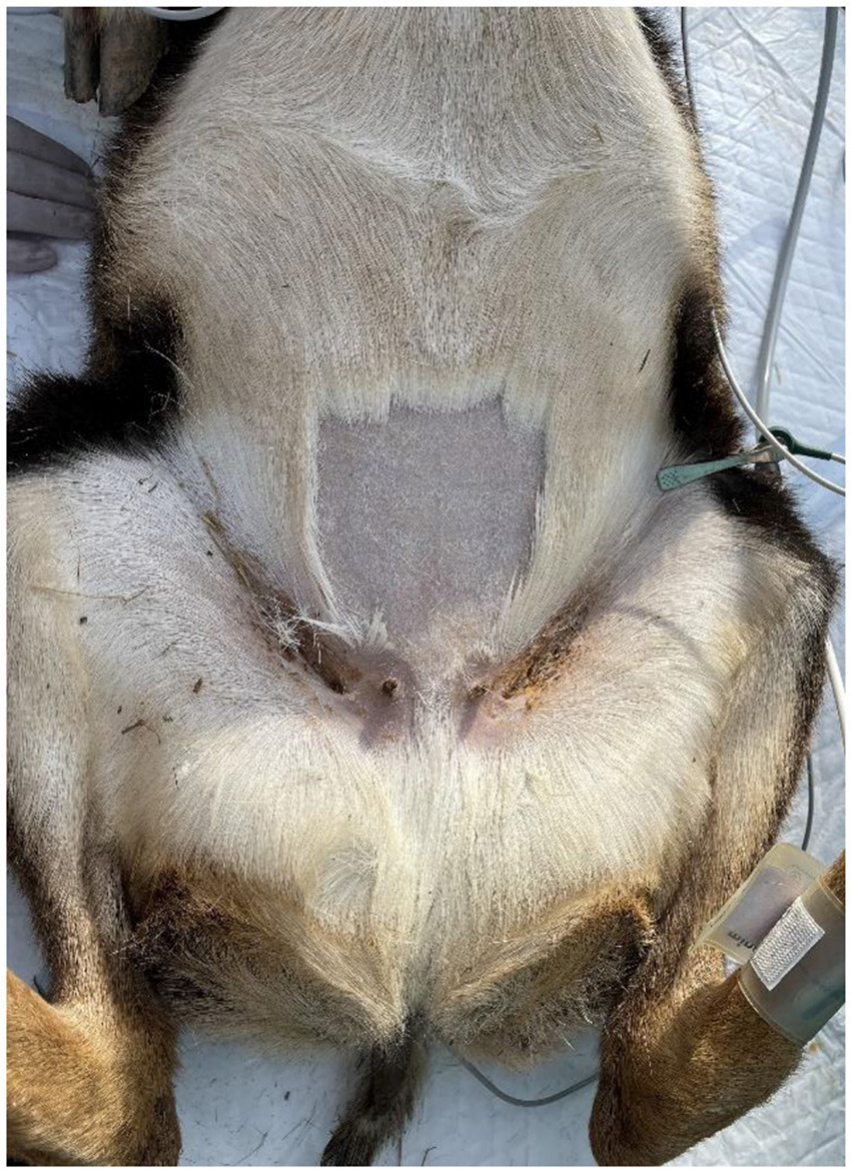
Surgical site to perform ovariectomy from the linea alba in a mouflon.

**Figure 5:**
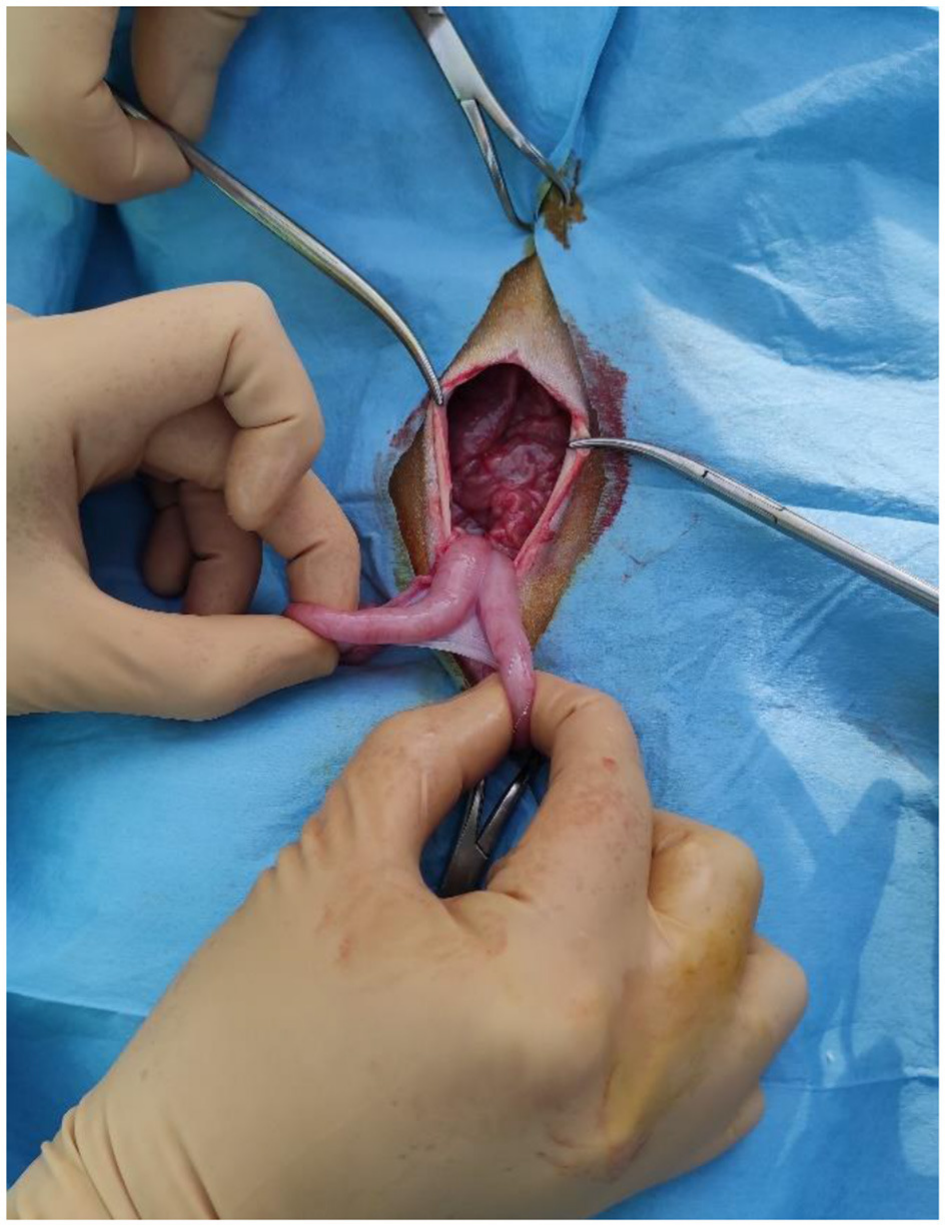
The uterus and the ovary of a mouflon exteriorized from the linea alba surgical site.

The area between the transverse processes of the lumbar and sacral vertebrae and from the last rib to the level of the tuber coxae, known as the paralumbar fossa, was clipped, shaved, and aseptically prepared. A vertical skin incision (about 6 cm long) was made on the left flank, midway between the last rib and tuber coxae, using a number 23 scalpel blade. The surgical procedure involved puncturing all muscular layers—the external and internal abdominal oblique muscles and transverse muscle—with a scalpel to gain surgical access. The muscle fibers were then separated down to the peritoneum, which was held with forceps, punctured with the scalpel, and cut with scissors. The surgeon used their fingers to grasp the uterus and locate the ovaries, which were then exteriorized (Figure 2). The ovary was removed using the Caiman^®^ (Caiman^®^ 5 non-articulated; Aesculap AG, Tuttlingen, Germany) vessel sealing device (handpiece 5 mm straight bite non-articulated jaw length 24 cm) by applying the clamp to the base of the ovary, at the level of the ovarian pedicle (Figure 3). Thereafter, signs of any hemorrhages from the remnant ovarian pedicle were observed. The same procedure was performed on the other ovary, following the contralateral uterine horn, through the same skin incision. After the ovaries were removed, the peritoneum and the abdominal transverse muscle were sutured together. A second layer of sutures was used to close the internal and external abdominal oblique muscles. Both layers were sutured using synthetic absorbable suture USP2 (Surgicryl^®^ Polyglycolic Acid PGA, SMI, Belgium). The subcutaneous tissue and skin were closed with simple interrupted sutures using the same suture thread. A total of 10 animals (group L) were ovariectomized via the linea alba approach (Figure 4). The surgical incision was made at the udder, ~10 cm from the umbilical scar, for a length of ~4 cm. The skin was incised and the linea alba was identified. It was raised, using a surgical clamp; an incision was performed using a scalpel and then enlarged with scissors. The surgeon grasped the uterus, using fingers, and exteriorized it, locating the ovaries (Figure 5). Following the aforementioned procedures, the ovaries were removed, and the abdominal wall was sutured using a continuous synthetic absorbable suture thread USP2 (Surgicryl^®^ Polyglycolic Acid PGA, SMI, Belgium). The subcutaneous tissue and skin were then closed with simple interrupted sutures using the same absorbable suture thread.

After the surgery, all animals were given a subcutaneous (SC) injection of trihydrate amoxicillin (Betamox LA 150 mg/mL, Vètoquinol Italia S.r.l., Bertinoro FC, Italy) at dose of 15 mg/kg and an IM injection of ketoprofen (Zooketo 100 mg/mL, Elanco Italia S.p.A., Milano, Italy) at a dose of 0.3 mg/kg for antibiotic and anti-inflammatory coverage during the postoperative period.

After the surgeries, all animals were placed in special cases to ensure a peaceful and safe awakening. The veterinary staff monitored the animals during this period. Once fully awake, the animals were housed in a small stable within the enclosure.

### 2.5 Intraoperative evaluations

According to De Carvalho et al. (20), for intraoperative nociception evaluation, the parameters of HR, RR, MAP, and T, with the data collected from the multiparameter monitor, were considered at five different time points:

- Surgical preparation (with the animal under general anesthesia) (T0);
- skin incision (T1);
- resection of the left ovary (T2);
- resection of the right ovary (T3); and
- end of surgery (application of the last stitch to the skin) (T4).

### 2.6 Postoperative evaluations

The veterinary staff observed all the animals post surgery at 1, 3, 5 12, and 24 h to evaluate any behavioral changes, such as reluctance to move, reduced feed intake, altered social interaction, and changes in posture. If pain symptoms appeared, the animals would receive another IM injection of 0.3 mg/kg ketoprofen (Zooketo 100 mg/mL, Elanco Italia s.p.a., Milan, Italy). The day after surgery, the animals were released into a large enclosure.

### 2.7 Statistical analysis

The data collected were entered into a database using an Excel spreadsheet, and the data analysis was performed with statistical software SPSS 19 (IBM, NY). The continuous variables were described with mean ± standard deviation (SD). The Shapiro–Wilk test and Bartlett's test were used to assess the distributional normality and homoscedasticity of the continuous variables; and a normalization model was set up for those variables that are non-normally distributed. The general linear model (GLM) for repeated measures was used for within-group comparisons using the least significant difference (LSD) *post-hoc* test, while Student's *t*-test was used for between-group comparisons. For all tests, a *p*-value of < 0.05 was considered statistically significant.

## 3 Results

All surgeries were completed, and no intraoperative or postoperative complications occurred. The data regarding the duration of surgery, anesthesia, and recovery are shown in Table 1. No statistical difference was observed between the groups for these parameters. During the surgeries, there was no increase in parameters of more than 25% compared to initial values, and thus, no animals were treated with a bolus of fentanyl as rescue analgesia. The HR, RR, T, SpO_2_, and MAP values, measured during the intraoperative procedures, are shown in Table 2. The HR trend is similar between the groups from T0 to T1. In group L, there is a rapid increase in HR values from T1 to T2. Statistical differences between the groups were observed at T2 and T3 (Table 2). Regarding the RR trend, no statistically significant differences were observed between the two groups. In group F, there was a statistically significant reduction between T3 and T4, while there were statistically significant differences between the times T0, T2, T3, and T4 *vs*. T1 in group L, and there was a slight increase in the values at T1 (Table 2). T had a reduction in both groups: statistically significant differences were observed within the groups, each time, as shown in Table 2. SpO_2_ and MAP values were very similar in both groups. No statistically significant differences were observed either between groups or between times (Table 2). As for pain evaluation after surgery, no animals needed further analgesic administration, and all of them were released the next day after surgery without any complications.

**Table 1:** Duration of surgery, duration of anesthesia, and recovery time of F (ovariectomized via the flank approach) and L (ovariectomized via the linea alba approach) group mouflons expressed as mean ± standard deviation (±SD).

**Groups**	**Duration of surgery (min)**	**Duration of anesthesia (min)**	**Recovery time (min)**
Flank (F)	18 ± 7.08	47.2 ± 15.26	14.6 ± 3.03
Alba (L)	17.8 ± 1.55	46.2 ± 8.28	15.6 ± 3.12

**Table 2:** Heart rate (HR), respiratory rate (RR), temperature (T), peripheral oxygen saturation (SpO2), and mean arterial blood pressure (MAP) values of mouflons in groups F (ovariectomized via the flank approach) and L (ovariectomized via the linea alba approach) expressed as mean ± standard deviation, at time T0: baseline; T1: skin incision; T2: left ovary manipulation and removal; T3: right ovary manipulation and removal; and T4: end of surgery (after application of the last stitch on the skin).

	**Group**	**T0**	**T1**	**T2**	**T3**	**T4**
HR (bpm)	F	94.1 ± 10.55	90.7 ± 7.69a	80.6 ± 15.36Ab	90.7 ± 15.34C	87.8 ± 19.3a
	L	93.7 ± 12.87a	88 ± 15.62ac	104.7 ± 4.03Bb	104.6 ± 8.72Dd	88.8 ± 16.68ac
RR (bpm)	F	30.9 ± 5.43	30.2 ± 5.47	31.8 ± 6.41	30.7 ± 4.37a	26.9 ± 3.93b
	L	29 ± 3.56a	33.6 ± 8.26b	30.3 ± 8.18a	29.2 ± 6.41a	26 ± 1.63a
T (°C)	F	39.05 ± 0.91a	39.16 ± 0.83c	38.54 ± 0.49de	38.42 ± 0.47bdg	38.10 ± 0.37bdf
	L	38.79 ± 0.46a	38.75 ± 0.79a	38.19 ± 1.06b	38.31 ± 0.83b	37.81 ± 0.26b
SpO2 (%)	F	98.3 ± 2.26	98.7 ± 1.83	98 ± 1.41	98.3 ± 1.49	97.7 ± 2.71
	L	97.7 ± 2.63	97.7 ± 3.13	98.7 ± 1.25	98.4 ± 1.51	97.8 ± 2.25
MAP (mmHg)	F	64.4 ± 19.36	61.7 ± 14.25	67 ± 22.11	68.4 ± 19.48	69.10 ± 10.97
	L	62.9 ± 4.25	61.4 ± 11.96	72.2 ± 17.42	72.7 ± 16.29	71.6 ± 27.23

In column: A, B: p < 0.01; C, D: p < 0.05. In the row: a, b; c, d; e, f; f, g: p < 0.05.

## 4 Discussion

Ovariectomy, which is widely used in field settings, has been widely used to control the density of wild ungulate populations and is known for its cost-effectiveness and long-term effects (21). Considering the above information, the purpose of this study was to compare the laparotomic approach at the flank with the approach via the linea alba as surgical techniques to perform ovariectomy in mouflons under field conditions to control their births. The duration of surgery, duration of anesthesia, and recovery time of the animals were considered for comparison. In addition intraoperative nociception, postoperative pain and complications were evaluated. Regarding the anesthesiological and analgesic protocol used in the field, gaseous anesthesia was employed to reduce the risk of “polmonite ab ingestis” from possible ruminal regurgitation. This anesthesia is not commonly used in the field, but excellent results were observed both during and after surgery in terms of the quality of awakening of the animals and the absence of complications. The protocol did not include the use of local anesthetics as the animals were placed under general anesthesia. Regarding the antibiotic coverage, a combination of different antibacterial molecules was used to extend the spectrum of action of these molecules, and a long-acting formulation was chosen as it was not possible to continue administration in the following days without further stressing the animals. Antibiotics were used in addition to an anti-inflammatory drug (ketoprofen) only after surgery to minimize the risk of hemorrhage and to control postoperative pain (22). Abubakar et al. (16) evaluated the differences between the surgical approaches using the flank and the linea alba in goats. The lateral access appears to be the most widely used technique for approaching the abdomen of small ruminants; it allows surgery to be performed under local anesthesia, although it cannot be used in this study, as they are wild animals. The advantages of using the lateral access in wild animals are the ability to remotely observe the surgical site and the lower potential risk of evisceration in case of wound dehiscence due to the overlapping arrangement of the oblique muscles of the abdomen (16, 23). The ventral approach is an alternative with few intraoperative and postoperative complications, as the linea alba is incised, which has a lower incidence of bleeding (16). The disadvantage of ventral laparotomy is related to the risk of evisceration in the event of surgical wound dehiscence (5). Moreover, the use of vascular dissection and coagulation devices allows a reduction in the surgical time and safety in tissue bleeding, as reported by Cicirelli et al. (18) and Lacitignola et al. (24). In this study, in both groups, Caiman^®^ (Aesculap—Tuttlingen) was used to perform the surgeries, and there were no intraoperative or postoperative complications. No statistically significant differences between the two groups were also found for surgical duration (~20 min), anesthesia duration (~50 min), and recovery time (~18 min). Therefore, both the techniques are considered effective and easy to perform. Regarding the intraoperative evaluated parameters, statistically significant differences between the two groups were found at T2 and T3 for HR values. Specifically, there was a rapid increase in HR, evident only in group L at T2, which was kept consistently high until T3. In the linea alba approach, increased tension was exerted on the ovarian pedicle to exteriorize the ovaries, which represents a nociceptive stimulus detectable by the increase in HR. However, it should be taken into consideration that these values do not exceed 25% of the baseline value of the same group at T0, indicating that the increases are within normal ranges such that rescue analgesia during surgery was not necessary. Regarding the intraoperative values of RR, T, SpO_2_, and MAP, no statistically significant differences were found between group F and group L. There is a lack of references in the literature on the assessment of postoperative pain in mouflons. In fact, it is challenging to assess antalgic attitudes in wild animals, so this pain parameter was evaluated only with the observation of behavioral changes. In both groups, no alteration was observed.

## 5 Conclusion

It can be argued that the two surgical approaches, taking into consideration the duration of surgery, the intraoperative parameters evaluated, and the intraoperative and postoperative complications, have not shown relevant differences to favor the choice of one over the other. It can be affirmed that the use of access surgery from the flank enables better monitoring of the healing of the surgical wound from a distance and prevents potential evisceration due to wound dehiscence. These aspects offer a major advantage in the management of wildlife.

## Data availability statement

The original contributions presented in the study are included in the article, further inquiries can be directed to the corresponding author.

## Ethics statement

This study was performed in accordance with the ethical-guidelines of the Animal Welfare Committee. Institutional Review Board approval of the study was obtained from the University of Bari Aldo Moro with approval number 20/2023. The studies were conducted in accordance with the local legislation and institutional requirements. Written informed consent was obtained from the owners for the participation of their animals in this study.

## Author contributions

LF: Data curation, Investigation, Writing – original draft, Writing – review & editing. AC: Conceptualization, Data curation, Investigation, Methodology, Writing – original draft. MB: Conceptualization, Data curation, Project administration, Writing – original draft. FG: Supervision, Writing – review & editing. FL: Supervision, Writing – review & editing. AR: Conceptualization, Investigation, Methodology, Project administration, Supervision, Validation, Writing – review & editing. VC: Conceptualization, Investigation, Methodology, Supervision, Validation, Writing – original draft, Writing – review & editing.
